# Gel electrophoresis separation and origins of light emission in fluorophores prepared from citric acid and ethylenediamine

**DOI:** 10.1038/s41598-019-50922-6

**Published:** 2019-10-11

**Authors:** Alina A. Kokorina, Artem A. Bakal, Daria V. Shpuntova, Alexandr Yu. Kostritskiy, Natalia V. Beloglazova, Sarah De Saeger, Gleb B. Sukhorukov, Andrei V. Sapelkin, Irina Yu. Goryacheva

**Affiliations:** 1 Saratov State University, Astrakhanskaya, 83, Saratov, 410012 Russia; 2 Ghent University, Faculty of Pharmaceutical Sciences, Department of Bioanalysis, Centre of Excellence in Mycotoxicology and Public Health, Ottergemsesteenweg 460, 9000 Ghent, Belgium; 3 Queen Mary University of London, Mile End Road, London, E1 4NS UK

**Keywords:** Organic chemistry, Nanoscale materials, Nanoscale materials

## Abstract

We investigated light emission of hydrothermally treated citric acid and ethylenediamine (EDA) with various precursor ratios using gel-electrophoresis. We show that this relatively simple approach can deliver significant insights into the origins of photoluminescence. We found that products of the synthesis consist of both positively and negatively charged species and exhibit large dispersion in electrophoretic mobility (i.e. charge-to-size ratio). We observed that despite the large dispersion of the reaction products the blue light emission is confined to discrete bands clearly identifiable in the gel. We demonstrate clear evidence that this emission originates from the negatively charged light molecular fraction with the highest mobility which shows no excitation-dependent light emission. This molecular fluorophore exhibits spectral characteristics similar to previously reported 1,2,3,5-tetrahydro-5-oxo-imidazo[1,2-a]pyridine-7-carboxylic acid (IPCA). Secondary gel electrophoresis run performed on the bands extracted from the first run indicates that no further separation takes place. On the basis of our experimental results, we conclude that relatively stable binding exists between IPCA and EDA-derived product. Thus, the products of the reaction contain IPCA both in molecular form and in complexes with EDA-derived products. We conclude that excitation-dependent emission is related to the fluorophore binding to the positively charged EDA-derived products with a positive charge.

## Introduction

Light emitting carbon nanodots (CNDs) have recently emerged as a new family of low dimensional light-emitting nanocarbon materials^1^. Compared to the more conventional inorganic light-emitting quantum dots (e.g. CdSe, CdS, Si and Ge), CNDs have clear advantages such as low environmental impact, low cytotoxicity, excellent biocompatibility, and tuneable surface functionalities. These appealing properties of CNDs suggest great opportunities for applications ranging from consumer electronics^1^, to light harvesting^2^ and biological cell imaging^3,4^.

The vast majority of CNDs today are produced via hydrothermal (HT) treatment – a process of conversion (typically in an aqueous solution under elevated pressure and temperature) of organic compounds – since it is a relatively easy, high yield method that can be easily adopted for, scaled and integrated into industrial production. However, there are major issues in the production of CNDs by this method such as sample heterogeneity and uncertainty about CNDs structure^1,5,6^. As a consequence, there exist significant challenges in understanding the relationship between structure and the origin of the light emission as well as in control of the synthesis products. In fact, in many cases, the structure of these systems has not even been unambiguously established. Today the body of research on hydrothermally obtained photoluminescent (PL) materials is vast but the picture is still complicated with a number of outstanding questions including the structure and its influence on the optical properties. Understanding the fundamental rules that govern emission in CNDs in relationship to their structure will create a breakthrough allowing fluorescent carbons to compete with the inorganic semiconductor quantum dots.

Fluorophores prepared from citric acid (CA) and amines have become some of the most popular systems due to relatively high quantum yield (QY) (reported values are 73%^7^, 67,5%^8^, 94%^9^, 80%^3^, 86%^10^, 76 and 66%^11^). It has been recently suggested^10–12^ that blue emission may, in fact, originate from molecular species: i.e. 1,2,3,5-tetrahydro-5-oxo-imidazo[1,2-a]pyridine-7-carboxylic acid (IPCA, in case of synthesis using CA and ethylenediamine, EDA) or 5-oxo-3,5-dihydro-2H-thiazolo[3,2-a]pyridine-3,7-dicarboxylic acid (using CA and L-cysteine) and 5-oxo-3,5-dihydro-2H-thiazolo[3,2-a]pyridine-7-carboxylic acid (using CA and cysteamine) formed during HT synthesis rather than from CNDs themselves. Schneider *et al*.^12^ studied the properties of samples synthesized from citric acid and three different nitrogen sources: ethylenediamine, hexamethylenetetramine, and triethanolamine and suggested that luminescence originates form citrazinic acid derivatives which may exist in solution and bound to CNDs. Recent spectroscopic characterization of CA-EDA derived products indirectly shows^13^ that CNDs may play no role in the blue emission ascribing the PL properties entirely to the IPCA molecules.

It is now clear that HT synthesis from CA and a variety of organic precursors results in a heterogeneous mixture that includes light emitting species^14–16^. This makes all more poignant the need for an efficient and simple methodology for separation of synthesis products and for characterization of individual fractions. Some success has been achieved using sucrose density gradient centrifugation^17–19^. However, this method is challenging because of a necessity of placing several different density sucrose layers into a flask while avoiding their mixing and extracting fractions from the sucrose matrix^14^. Another method for separation of CNDs fractions is size-exclusion chromatography^10,20,21^. The disadvantage of this method is strong dilution of the initial material and a possibility of loss some species on the solid matrix. At the same time, in the recent work^22^ utilizing gel electrophoresis for separation of CNDs prepared from CA and EDA, authors suggest contributions to the PL from aromatic domains (PL and absorption at longer wavelength) and molecular fluorophores (PL and absorption at shorter wavelength). Authors further suggest^22^ that the molecular fluorophore is most likely embedded into CNDs acting as seeds for growth of aromatic domains. Hence, gel electrophoresis has been shown as a promising method for CND separation and the effect of synthesis time on the optical properties of CNDs has been investigated.

In this work we study effects of the CA:EDA precursor ratio on the optical properties of fluorophores prepared by HT synthesis and investigate stability of these products by utilizing two-stage gel electrophoresis separation. Gel electrophoresis was selected for it allows segregation of components by charge-to-size ratio since HT-prepared products may exhibit some variations of the surface charge due to the surface groups. This method also provides a clear visual evaluation of separated compounds obtained as a result of the HT synthesis. Furthermore, it requires only a small amount of sample (20–30 µl) and separated components can be relatively easily physically extracted by cutting the gel and washing out samples or using freeze-drying method^22^. HT synthesis from CA and EDA was selected because it results in fluorophore with high PL QY and samples prepared by this method has already been widely studied^12,23^. In order to optimize the synthesis, we analysed data from literature and identified conditions, which typically result in the formation of carbon nanostructures. According to the literature, the temperature of HT synthesis for CA:EDA nanoparticles is usually below 200 °C (70 °C^24^, 100 °C^23^, 110 °C^25^, 160 °C^9^, 180 °C^26^, 200 °C^10^). In our case temperature of 200 °C and 3 hours of HT treatment was chosen to complete conversion of precursors to final products and to facilitate direct comparison with the recent work^14^. Samples with the precursor ratio of CA:EDA 1:0.5, 1:1, 1:1.5, 1:2, 1:3, 1:4, 1:6 were synthesized and their optical properties were examined. For the more detailed analysis the sample with a slight excess of EDA (molar ratio CA:EDA 1:1.5) was used to ensure conversion of CA (see Fig. S1), since for IPCA synthesis CA:EDA stoichiometric ratio is equal to 1:1^12^.

**Figure 1:**
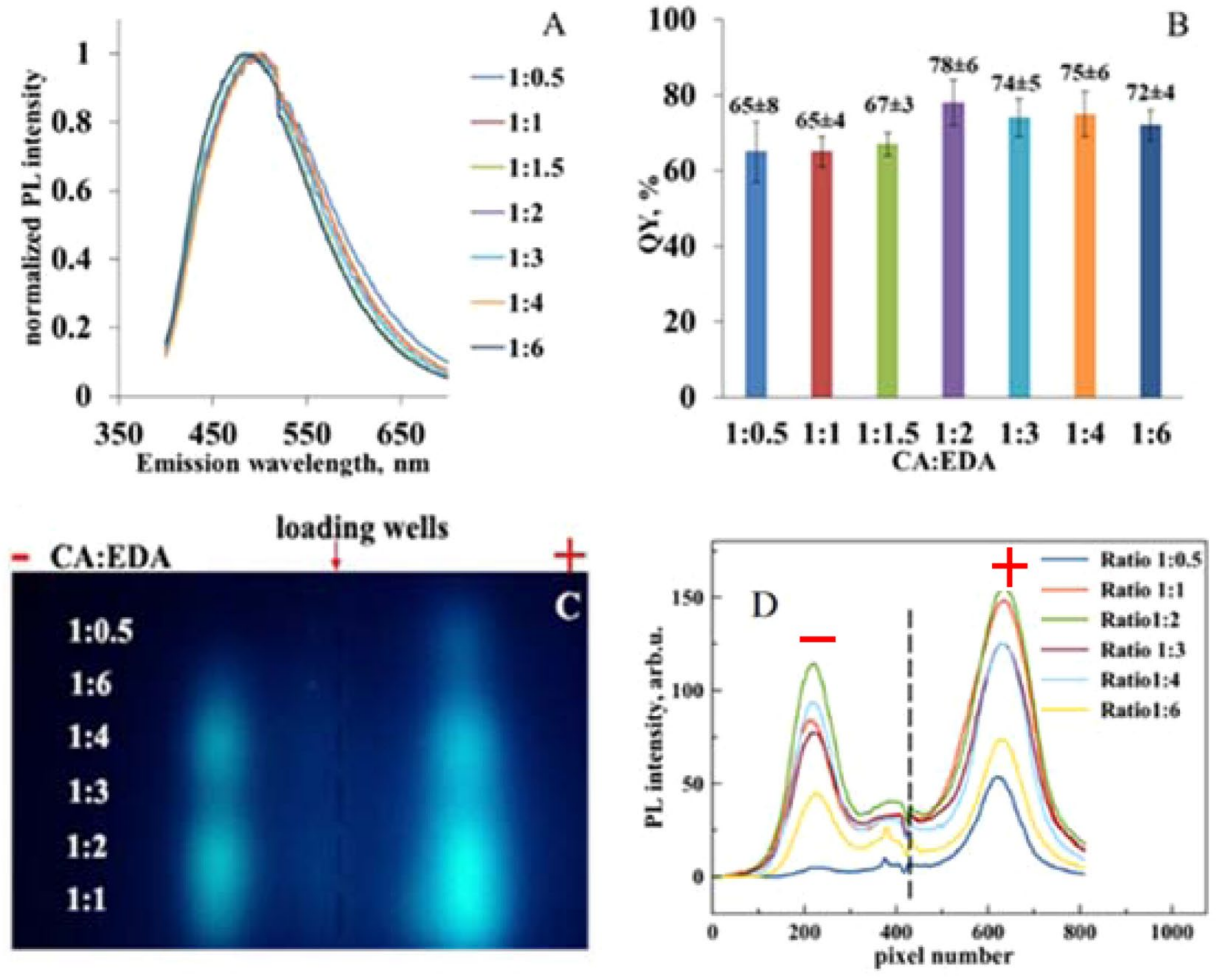
Normalized PL spectra (λ_ex_ = 350 nm) for the samples with different CA:EDA ratio (**A**), relative QY data (**B**), UV-excited image of gel-separated species (**C**) and the corresponding profile intensity distribution (**D**). Loading wells position is marked with the red arrow.

First, we examined the effect of CA:EDA ratio on the optical properties of the obtained fluorophores. We found that all samples show similar optical absorption signal (Fig. S2) and normalized PL spectra (with 350 nm excitation) for all precursor ratios (Fig. 1A) with maxima at 450 nm. We further found that QY of the observed emission does not depend on the precursors ratio to any significant extent (Fig. 1B) with the highest QY obtained for CA:EDA = 1:2 that is consistent with the data reported previously^10^.

The UV-excited light emission image of the gel electrophoresis is shown in Fig. 1C with the intensity profile of obtained lanes for each CA:EDA ratio in Fig. 1D (see more detailed analysis in Fig. S3). Here all initial samples were diluted 1000 times to avoid possible PL signal quenching due to self-absorption.

The main finding, which is clear from Fig. 1C,D is presence of the same types of luminescent products. All tested CA:EDA ratios give both positively and negatively charged PL bands (even though emission of negatively charged PL bands for 1:05 ratio is very weak). Electrophoretic mobility of both positive and negative bands (distances from the loading wells) does not depend on the CA:EDA ratio, indicating the same charge-to-size ratio for all negative as well as for all positive fluorophores. The lane for the product, obtained at CA:EDA ratio 1:0.5 is slightly different from the others. It exhibits the lowest PL intensity and the ratio of positive-to-negative bands PL intensity is much higher (~11) than for all other samples (<2) (Fig. S3C). This suggests a relatively low yield of the fluorophore with a net positive charge when the reaction is “EDA-starved”. Our data also suggests that the optimal CA:EDA ratio for the fluorophore production is between 1:1 and 1:2 (the reaction scheme gives 1:1 ratio, see Fig. S1). Hence, a sample with CA:EDA at 1:1.5 ratio was chosen for detailed investigation of fluorophores obtained in HT synthesis.

**Figure 2:**
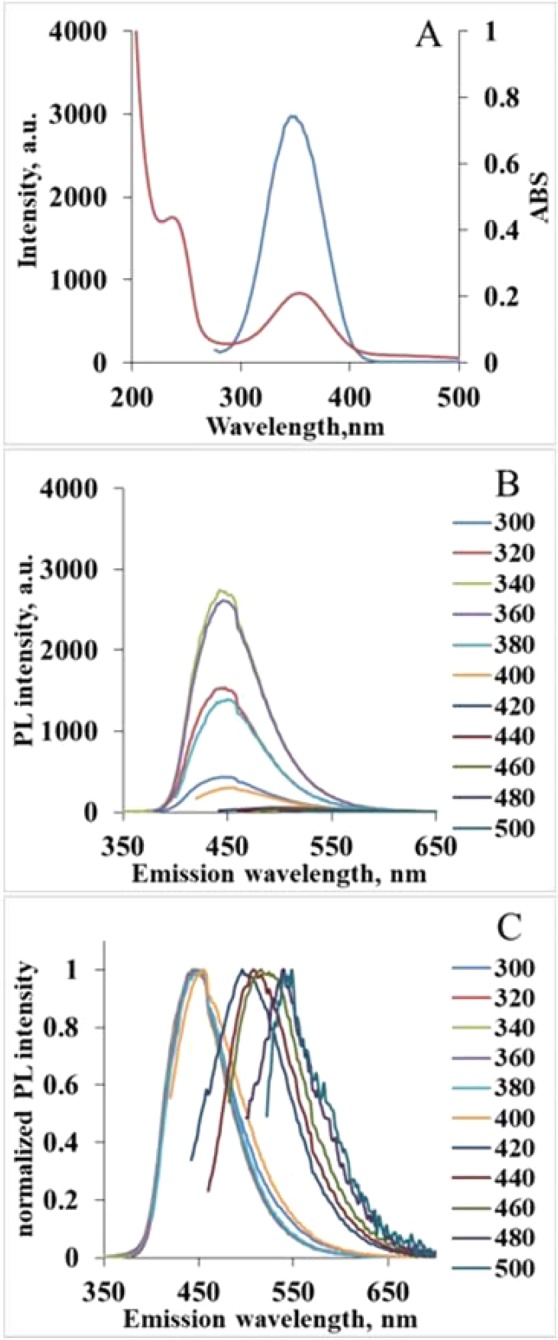
Spectra of HT treated CA:EDA, 1:1.5 solution: absorbance (red) and excitation (blue, measured at λ_fl_ = 450 nm) spectra (**A**); PL spectra (**B**) and normalized PL spectra (**C**) at different excitation wavelengths.

The summary of absorption, excitation and emission spectra of the sample with 1:1.5 ratio is shown in Fig. 2. Excitation in 300–400 nm range results in emission with a maximum at 450 nm. The observed Stokes shift is around 100 nm and one can see a clear symmetry of excitation and emission peaks (Fig. S4), which is typical for organic fluorophores. Obtained spectral characteristics (Figs S2 and S4) are matching those of IPCA literature data^20,23^. Excitation in 420–500 nm range results in a typical for CNDs excitation-dependent emission (Fig. 2C) with much lower emission intensity).

To explore the structure of samples, we performed ^1^H-NMR, ^13^С-NMR, HSQC, HMBC analysis. The spectra we obtained confirm presence of IPCA fluorophore and are in agreement with previously published findings by Song^10^. Two methylene units of the aliphatic fragment of the molecule appear as triplets at 3.79 ppm and 4.16 ppm. The signals of the two protons of the pyridine ring are located at 5.94 ppm and 6.05 ppm. Broad singlet in the area of 7.75–8.00 ppm correlates to the NH group (Fig. S5A). The signals at 3.5 to 2.0 ppm correlates with polymeric structure from CA and EDA. In the ^13^С-NMR spectrum, the characteristic signals are: the carbon signal of the carboxyl group at 172.48 ppm, the signals of carbon atoms of the CH_2_ groups are at 45.03 ppm and 47.60 ppm. The carbon of the pyridine ring appears at 89.16, 105.03, 149.92, 157.15 and 164.87 ppm. (Fig. S5B). The HSQC, HMBC also confirm the fluorophore structure (Fig. S5C,D). This suggests that IPCA is present in the reaction products as a separate molecule, rather than being integrated into larger polymer structures.

**Figure 3:**
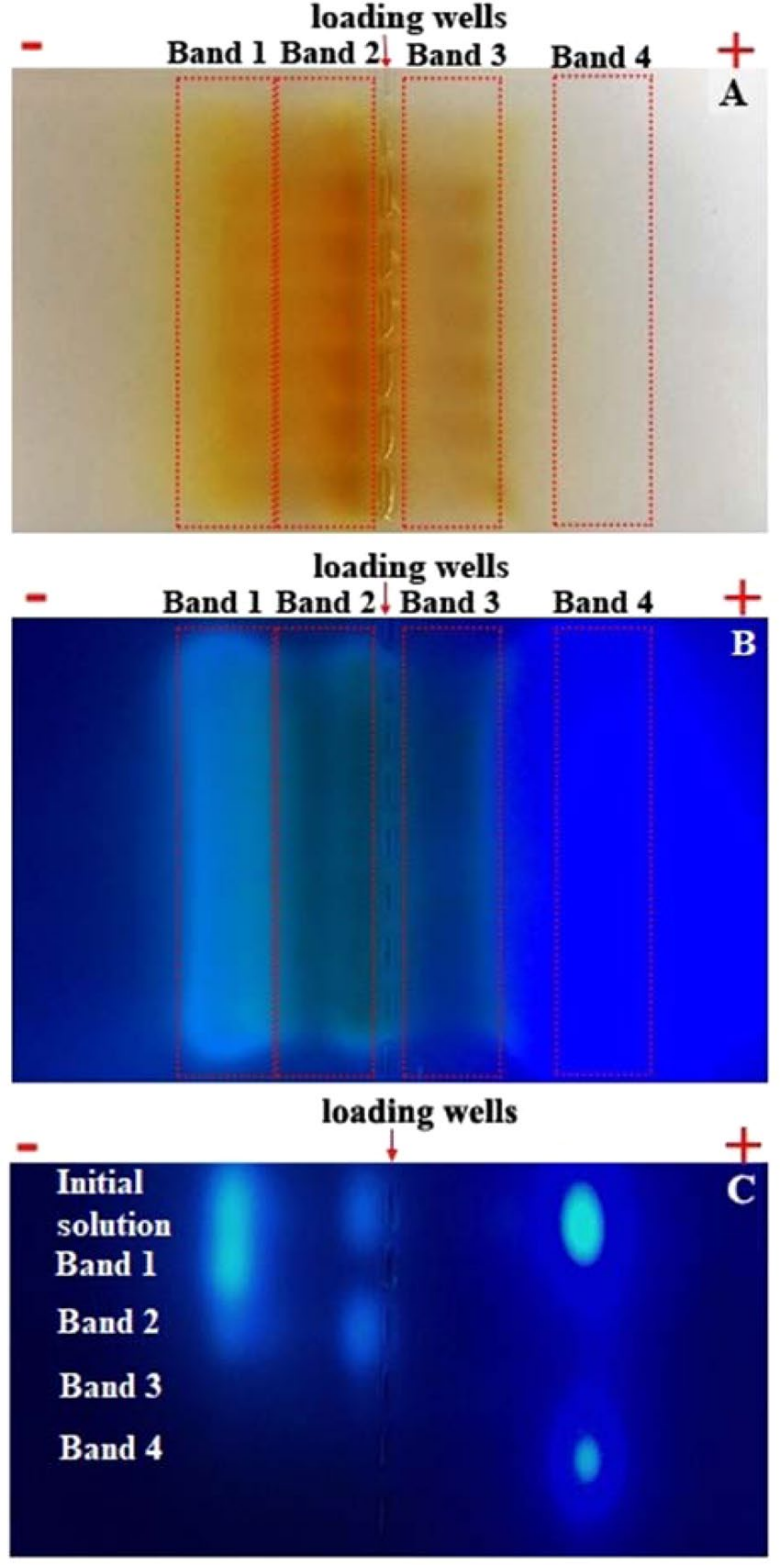
Gel electrophoresis separation of HT treated initial solution (CA:EDA = 1:1.5): white light (**A**) and UV-light (**B**) photos. The secondary electrophoresis run of electrophoretically-separated bands 1–4 under UV light (**C**). For comparison, the top lane of 10 times diluted initial solution is also presented, which corresponds to the high contrast result (**B**). Full (non-cropped) parts of the gels are shown in all cases.

Results of gel electrophoresis separation of this sample are shown in Fig. 3A,B. Eight wells were loaded with portions of the same sample to test repeatability of the procedure. It is immediately clear that several bands (designated as band 1–4) can be identified (based on the white light and UV light imaging) both on the positive and the negative sides of the lanes (see Fig. 3A,B). Each of the bands (1–4) may, of course, contain a number of molecular species, but they do not seem to have an impact on electrophoretic mobility. Here a non-diluted sample was used for better visualization of band 3 with low PL intensity (which results in saturation in the region of band 4 in the image under UV excitation, see Fig. 3B). From the white light image (Fig. 3A), it is clear that the sample consists of brown-coloured species (both positively and negatively charged) of different charge-to-size ratio with larger (and darker) particles located closer to the loading wells. UV-excited blue light emission can be clearly observed confined to the discrete bands (Fig. 3B), with the most intense emission originating from the negatively charged fluorophore farthest away from the loading wells (designated as band 4 – the species with the largest charge-to-size ratio).

Overall, the observed PL emission pattern in the gel electrophoresis is similar to the one reported^22^ for samples with 1 hr synthesis time at 200 °C with 1:1 CA:EDA ratio (also showing distinct emission bands). The results of the transmission and emission measurements (Fig. 3) suggest that a fluorophore with a large charge-to-size ratio is present among the synthesis products. The nature of brown-coloured particles has been previously established as a mixture of nanosized polycyclic aromatic carbons and of more complex polymeric carbon-based structures^6,27^ with average size on the scale of ~1 nm^10,14^. It has been reported^22^ that the average size of all CNDs in the reaction product is similar (~1 nm) and is independent of the synthesis time. From this authors concluded that the observed separation in the gel pattern is chiefly due to particle charge. In such a context, the relatively transparent gel region with the strong emission (region designated “−4” in ref.^22^) may indicate a relatively small amount of sample in that region with a large (compared to the rest of the sample) value of QY. Alternatively, it may suggest that the region contains large amount of transparent small molecular-like fluorophore not detected by TEM/AFM and hence not included in the reported particle size distribution. This is consistent with presence of IPCA-like fluorophore reported in these systems^10–12^. The latter interpretation is certainly consistent with the absorption data (Fig. S2) that point to a system with a large (~3.5 eV) HOMO-LUMO gap. At the same time, the brownish colour of the EDA-derived product suggests that the corresponding energy gap (HOMO-LUMO) is certainly below ~2.2 eV  in these systems.

**Figure 4:**
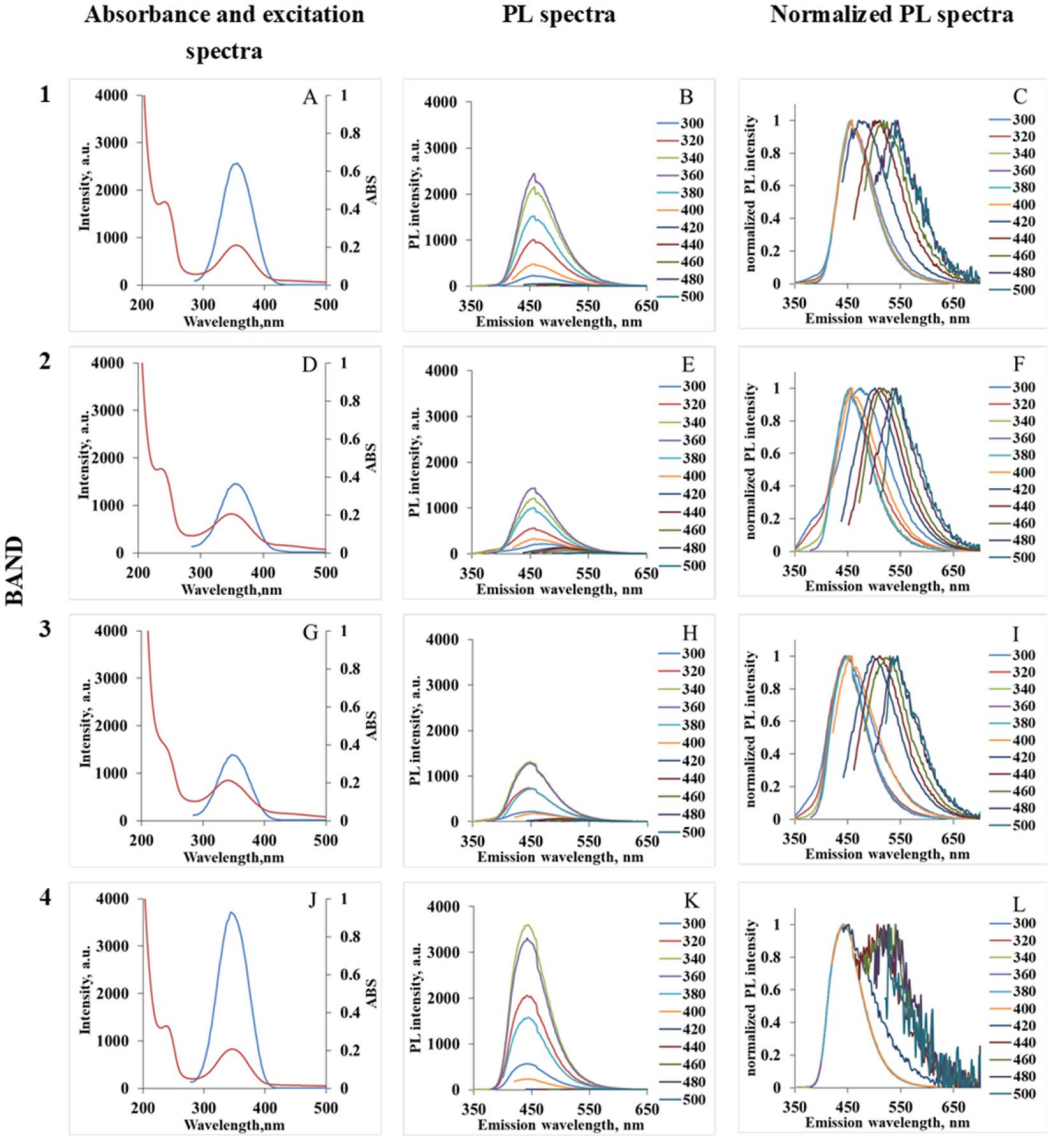
Absorbance (red) and excitation (blue) spectra (first column; spectra (**A**,**D**,**G**,**J**); PL spectra (second column; spectra (**B**,**E**,**H**,**K**) and normalized PL spectra (third column; spectra (**C**,**F**,**I**,**L**) of extracted bands. The first row presents data of band 1; the second row of band 2; the third row of band 3 and the fourth row of band 4.

In order to differentiate between the two possibilities mentioned above (smallest size *vs* smallest amount of brownish coloured species) for the nature of band 4, we carried out optical measurements on each of the bands extracted from the gel. Absorption, excitation, PL and normalized PL data for bands 1–4 are shown in Fig. 4. The similarity in the absorption and excitation spectra for all bands again suggests a matching fluorophore system is responsible for the blue light emission in all cases. The brightest PL is obtained for band 4 with a maximum at 450 nm (for excitation at 300–400 nm, Fig. 4K), which is well-documented for IPCA^10,12^. The behaviour of PL spectra of this band (Fig. 4K) is significantly different from the rest of the bands, as they show no excitation-dependence (PL signals with excitation above 400 nm are at the level of noise, see normalized data in Fig. 4L).

**Figure 5:**
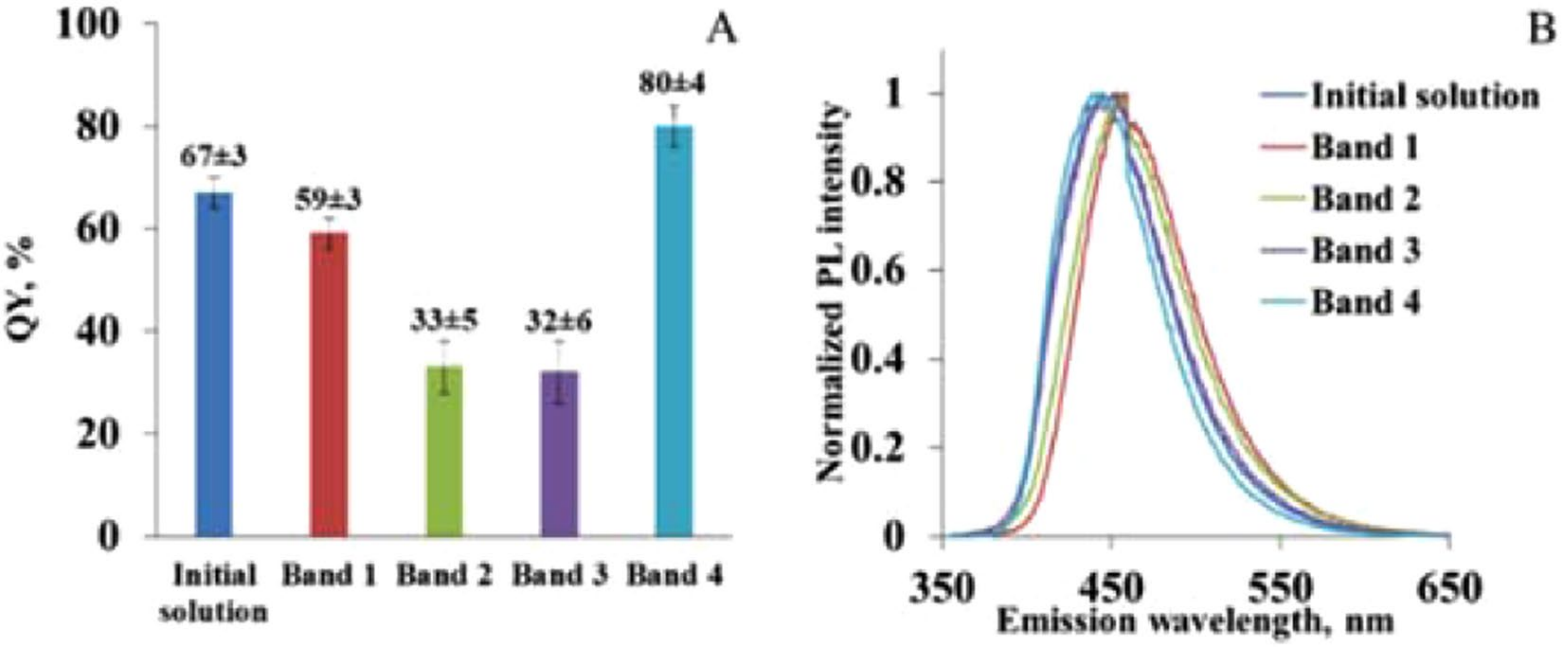
QY (**A**) and normalized PL spectra (**B**) of initial solution and bands 1–4 at λ_ex_ = 350 nm.

Furthermore, one can see in Fig. 3A that emission from the band 4 originates from the region, that is transparent (i.e. has no obvious brown coloration) and farthest away from the loading wells. QY measurements for each of the bands show (see Fig. 5A) that particles with the largest value of QY of around 80% are found in the band 4. This is also the band with the strongest emission intensity. This, together with the data in Fig. 4L (that show no excitation dependence of the emission) leads us to the conclusion that band 4 contains a transparent fraction of molecular species (IPCA or IPCA-like) responsible for PL in the blue spectral range. Thus, we conclude that emission associated with band 4 comes from the species with the largest charge-to-size ratio and the smallest fluorophore – most likely IPCA – having negative charge as a result of dissociation and hydrogen ion loss. Our analysis also shows that all other bands (bands 1–3) show excitation-dependent PL emission typical for CNDs reported before^5,11,28^. At the same time, one can see (Fig. 4A,D,G,J) that absorption and excitation spectra for the blue emission (excitation range 300–400 nm) for all bands are nearly identical, suggesting similar excitation and emission pumping mechanism in that wavelength range.

The next (after band 4) most intense PL originates form band 1 (Fig. 4B). The QY for this band is relatively high (59%) and the spectral characteristics of the main (strongest) peak is similar to IPCA. The excitation-dependent PL intensity (excitation above 400 nm) is relatively low. It is peculiar that this fluorophore system is positively charged, while for IPCA only negative charge is possible due to deprotonization. This suggests the contribution of positively charged amino-group contained in EDA and EDA-derived products (unlike polyaromatic-like compounds, which are neutral, as was suggested before^14^) into the brown colour of the band and into excitation-dependent PL at longer wavelength.

Band 2 shows the highest amount of brown coloured material, the highest contribution of excitation dependent PL (Fig. 4E), positive charge and low mobility. This suggests a higher amount of positively charged EDA-derivatives bound to the IPCA-like molecular fluorophore. Finally, band 3 shows the lowest PL intensity with lowest QY, negative charge, and low mobility, again allowing to suggest molecular fluorophore binding with the EDA-derived product. Negative charge demonstrates higher amount of IPCA-like molecular fluorophore compares to EDA-derived brown product. This is further confirmed by the ^1^H-NMR data for bands 1–4 present at Fig. S7. It is important to note that the spectrum for band 4 contains the signals corresponding to IPCA structure and shows no signals for larger polymer structures.

The question that remains is whether IPCA-like fluorophore and EDA-derived product form strong complexes during HT treatment with a fixed ratio between components or there is a dynamic equilibrium of complexes with different components ratios. In order to assess the strength of molecular fluorophore and EDA-derived product binding, we carried out further detailed investigations of bands 1–4 of the sample with 1:1.5 CA:EDA ratio. Bands 1–4 were cut out from the gel, samples extracted, suspended in water and subjected to secondary gel electrophoresis run. As a reference, a diluted (10 times to avoid saturation) initial solution was used in the same run. Secondary electrophoresis run shows that no further separation takes place (see Fig. 3C): each band (separated in the first run) was reproduced as a single band at the same position (as in the initial run) in the gel. Thus, only the initial sample gives several discrete bands (top lane in Fig. 3C). The same position (and so the same electrophoretic mobility) of the bands in first and in the second runs indicates retention of charge-to-mass ratios and no obvious further dissociation of fluorescent complexes or dynamic equilibrium between them. Presence of single band only in the second run shows that all four separated initial fluorophore complexes are stable and are not dissociate any further into molecular fluorophore and EDA-derived fraction.

Thus, we conclude that the emission in bands 1–3 originates from the complexes of positively-charged EDA-derived brown-coloured products (with excitation-dependent emission) and negatively-charged IPCA-like molecules. The different ratio of EDA-products and IPCA like molecules is responsible for different charge and size of the complexes, while the species surface charge influences the QY of the obtained bands. Band 4 has the highest QY (80 ± 4%) with a negative charge and also the highest charge to mass ratio. Band 1 has QY around 67% with a positive charge. Bands 2 and 3 have a positive and negative charge and QYs are around 32% and 33%, respectively and the least charge to mass ratio. Furthermore, binding in complexes is sufficiently strong to sustain secondary electrophoresis runs. The exact ratio of the brown-coloured EDA-derived products and molecular fluorophore for bands 1–3 is unclear at this point, but what is evident from the lanes observed in gel electrophoresis (Fig. 3A) is that both positively and negatively charged complexes are present and that amongst positively charged fraction there is a significant dispersion by charge-to-size ratio.

Thus, the data indicate that a single type of negatively charged IPCA-like fluorophore is responsible for the light emission at around 450 nm, while emission from the positively charged species originates from the fluorophore tethered to the brown-coloured reaction products that are most likely EDA-derived. Discrete nature of PL bands both in this and in previous^22^ work is related with some sort of complexes of the molecular fluorophore and the EDA-derived product and requires further investigation. Importantly, the data in Fig. 1C,D (and in Fig. S3C) indicate that very little positively-charged fluorophore is produced when reaction is “EDA-starved”. This suggests that by varying the precursor ratio and time (and perhaps temperature) of HT treatment one can tune to some extent the composition of the produced fluorophore system.

Our experiments with “EDA-starved” mode suggest that this fluorophore – most likely IPCA – is unlikely to act as a seed for growing larger particles^14^. Indeed, using 1:0.5 CA:EDA ratio results in a single strong emission band (see Fig. 1C) with negative charge and molecular-like emission, while increasing the fraction of EDA results in appearance of further positively-charged emission bands with increased contribution of excitation-dependent emission. This suggests that IPCA and brown-coloured fractions are produced in parallel followed by binding between positively and negatively charged reaction products. In summary, the results reported here clearly show that gel electrophoresis is an excellent tool to study a mixture of products in systems prepared by HT synthesis route since it results in physical separation of reaction products for subsequent analysis. Furthermore, it can be used effectively to separate fractions with excitation-dependent and independent emission, while providing a route to linking fluorophore yield and its properties to the synthesis conditions and precursor selection. It has been pointed out in a number of reviews^1,6^ that despite significant progress in synthesis and characterizations of CNDs, uncertainties in size distribution, chemical composition and origins of PL significantly delay further progress. The next step in the development of the CNDs research must be associated with a continuous shift towards synthesis of CNDs engineered for the purpose. This requires detailed understanding not only of a chemical composition of the final product and its optical properties, but an ability to establish a clear relationship between precursors, conditions of reaction and physical properties of produced CNDs. We believe that because of its sensitivity to both charge and size gel electrophoresis in nanoscale systems can become an invaluable tool to address these challenges.

## Supplementary information

Gel electrophoresis separation and origins of light emission in fluorophores prepared from citric acid and ethylenediamine
