# Supplementary material for: Gel electrophoresis separation and origins of light emission in fluorophores prepared from citric acid and ethylenediamine
Source: Sci Rep. 2019 Oct 11;9:14665. doi: 10.1038/s41598-019-50922-6 (PMC6789027; doi:10.1038/s41598-019-50922-6)
Supplement: Supplementary file 1 — Gel electrophoresis separation and origins of light emission in fluorophores prepared from citric acid and ethylenediamine [file 41598_2019_50922_MOESM1_ESM.docx]

Gel electrophoresis separation and origins of light emission in fluorophores prepared from citric acid **and ethylenediamine**

Alina A. Kokorina, Artem A. Bakal, Daria V. Shpuntova, Alexandr Yu. Kostritskiy, Natalia V. Beloglazova, Sarah DeSaeger, Gleb B. Sukhorukov, Andrei V. Sapelkin, Irina Yu. Goryacheva

**SUPPORTING INFORMATION**

Experimental section

CA, EDA, TRIS borate EDTA buffer, quinine hemisulfate salt monohydrate and Agarose NA were purchased from Sigma Aldrich. All reagents were of analytical grade. Double-distilled water was used in all of the experimental processes. The absorbance spectra for all samples were collected on Shimadzu UV-1800 UV–vis spectrophotometer. PL and excitation spectra were measured with Cary Eclipse spectrofluorimeter (Agilent Technologies, Australia). ^1^H-NMR, ^13^С-NMR, HSQC, HMBC spectra were recorded at 20–25 °C on a Varian-400 spectrometer (400 and 100 MHz, respectively; Agilent Technologies, Santa Clara, CA, USA), using D_2_O as a solvent and 4,4-dimethyl-4-silapentane-1-sulfonic acid as an internal standard.

For HT synthesis CA and EDA precursors with different molar ratios CA:EDA (1:0.5, 1:1, 1:1.5, 1:2, 1:3, 1:4, 1:6) were used. CA initial concentration was 1 M for all experiments. CA was dissolved in double-distilled water, stirring on a Vortex mixer for 1 minute and EDA was carefully dropwise added to the solution. The reaction mixture was stirred for 3 min and 3 ml was placed to the glass cup and then transferred to Teflon cup with hard fixed cover, and heated in a stainless-steel autoclave at 200˚C for 3 hours. Then the autoclave was cooled at RT and the final dark-brown solution was centrifuged (15 min, 7500 rpm) to remove any big pieces. The pH values of the final solutions are 2.5, 4, 5, 8.5, 10, 10, 12 for the supernatants, obtained with CA:EDA ratio 1:0.5, 1:1, 1:1.5, 1:2, 1:3, 1:4, 1:6, respectively.

Solution after centrifugation was separated by horizontal gel electrophoresis. Agarose (2%) solution was prepared in TRIS borate EDTA buffer: 1.6 g of Agarose was placed in a thermally-resistant glass, then dissolved in 78.4 ml of the buffer and heated at 250˚C to transparency solution. The solution was cooled at RT to 50˚C. The warm agarose solution was placed to the special form for gel electrophoresis, avoiding bubbles, and hardened for 30 min. For the analysis aliquots (20μl) were placed to the loading wells. The gel electrophoresis process was performed at 150V, 300 mA for 30 min in the same buffer.

The separation result was controlled under the day light and under UV excitation (UV lamp, λ_ex_ = 365 nm). For studying of separated species, the luminescent bands on gel surface were cut from the gel and shredded into small pieces. These pieces were placed into a plastic vial, 10 ml of double-distilled water was added and the mixture was stirred in a Vortex mixer for 7 minutes to extract luminescent species. The solution after extraction was centrifuged for 10 min at 7500 rpm to remove agarose fragments and uplayer solution containing luminescent species was collected for further experiments (optical studies, ^1^H-NMR analysis and the second gel electrophoresis run).

QY was measured through a relative method with quinine sulfate in a 0.1M sulphuric acid as a reference. The 350 nm absorbance maximum and optimal excitation wavelength were used. The QY was calculated according to the following equation:

**QY*_x_* = QY*_ST_* (I*_x_* / I*_ST_*)*(A*_ST_*/ A*_x_*)*(η^2^*_x_* / η^2^*_ST_*)**

where QY is quantum yield, I is PL integral intensity, A is absorbance, and η is the refraction index, while subscripts X and ST label the sample and the standard, respectively.

Image analysis has been carried out using Fiji - an open source image processing package^1^.

**
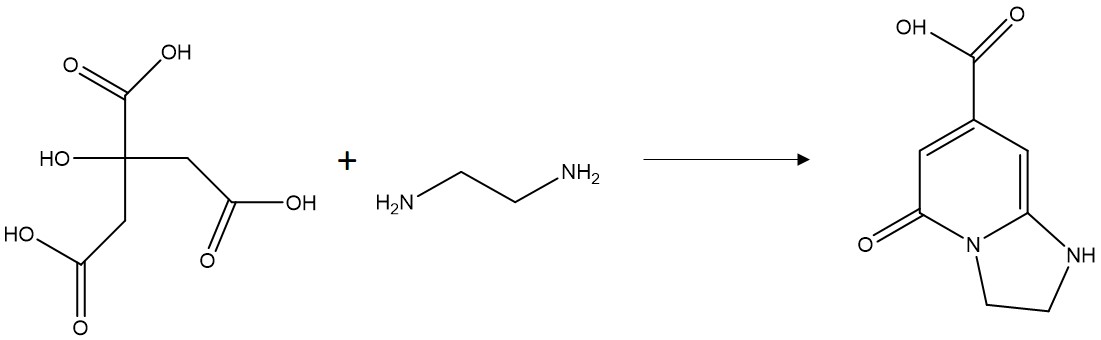
**

Fig. S1. Formatting scheme of IPCA molecule

**
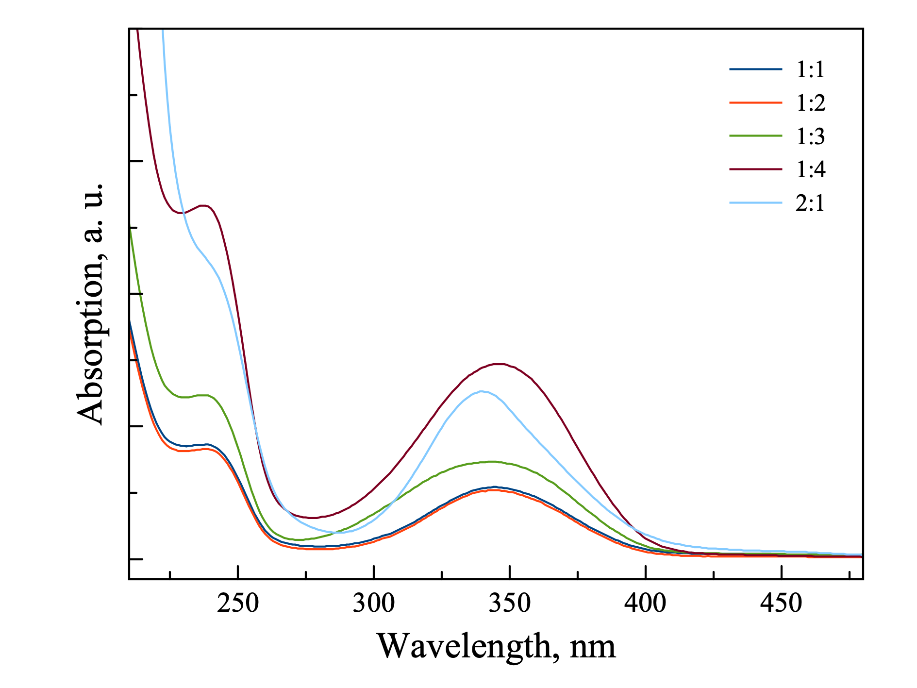
**

Fig. S2. Optical absorption in as-prepared samples as a function of CA:EDA precursor ratios.


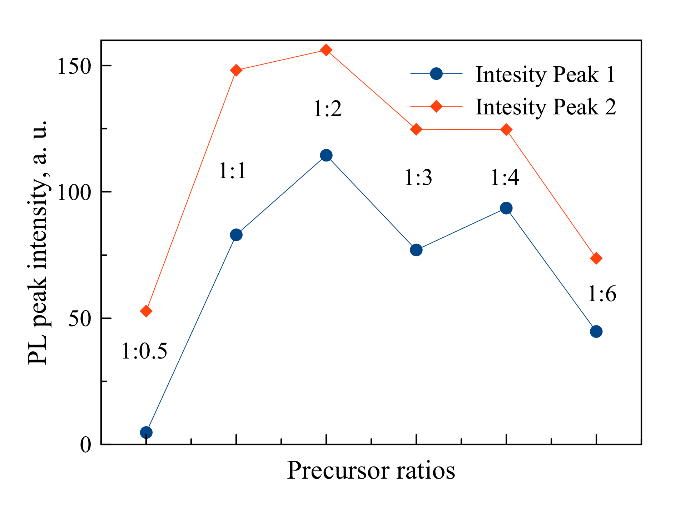

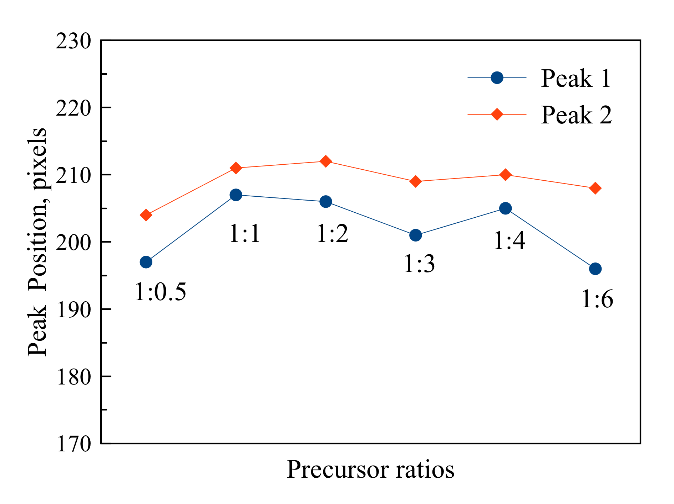

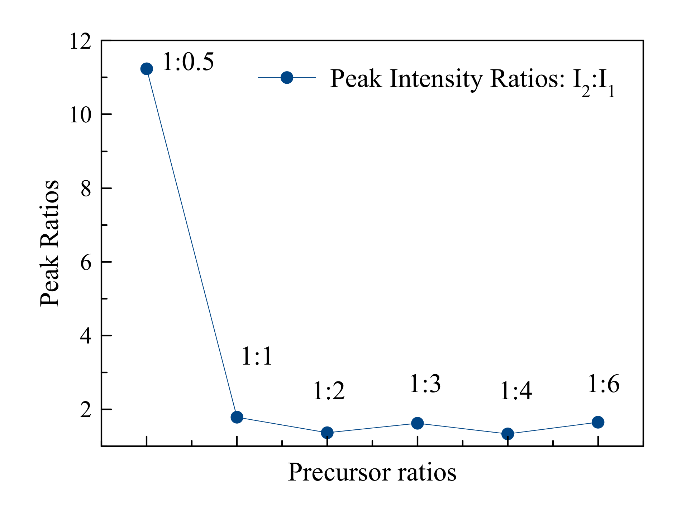

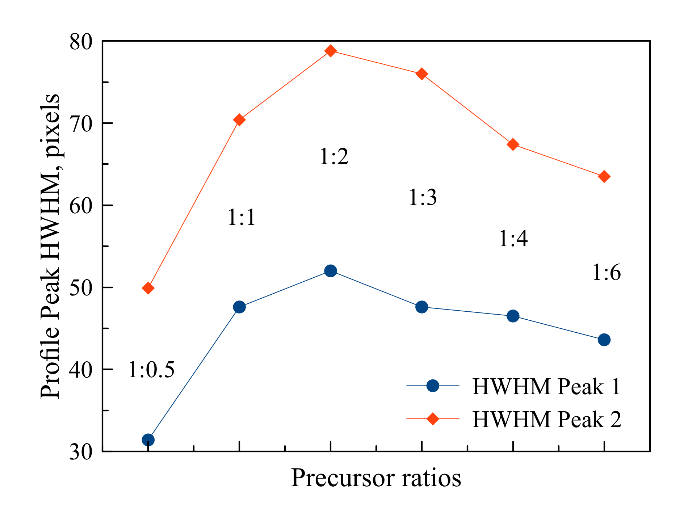


C
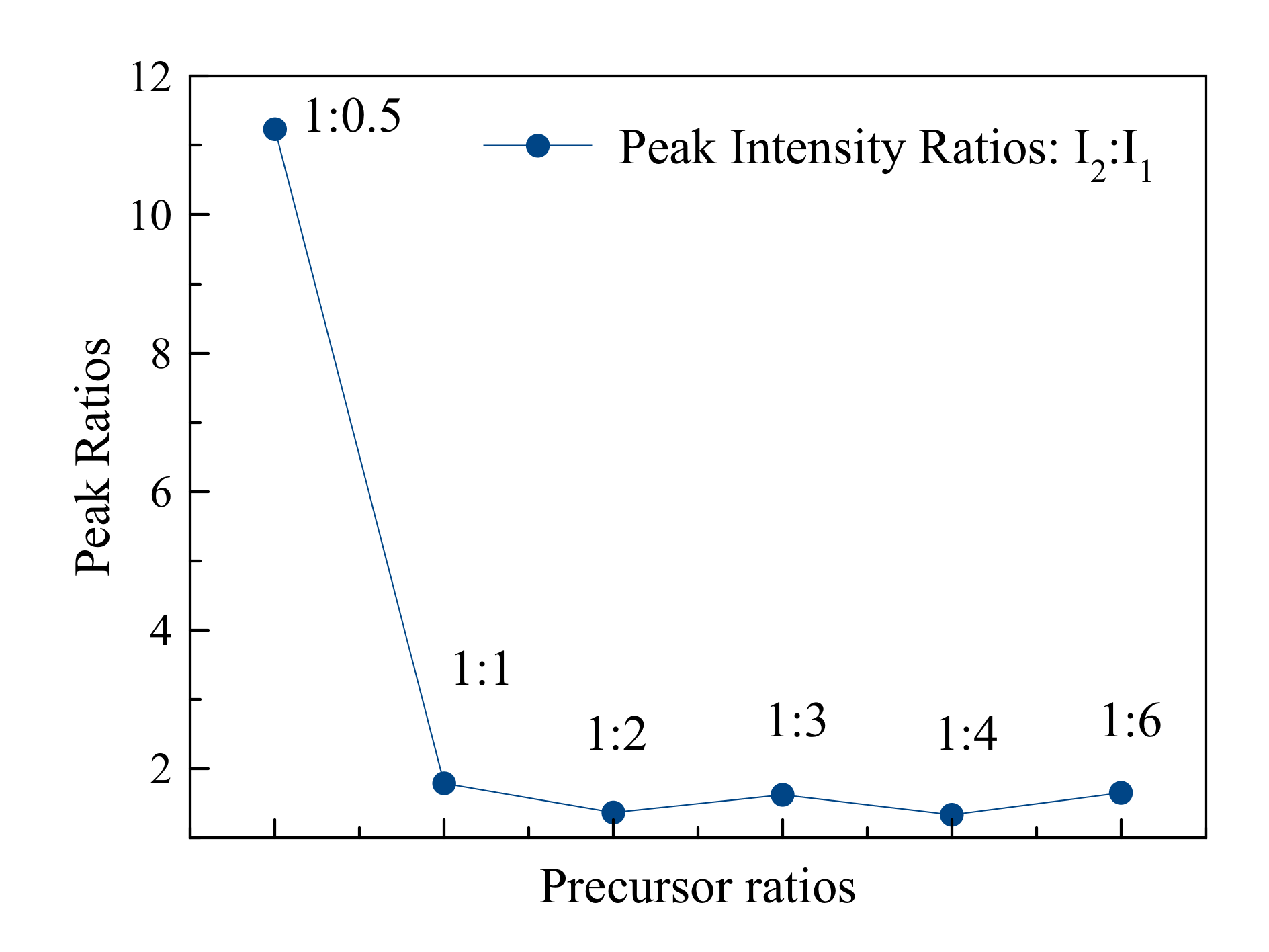


D

A

B

Fig. S3. Profile analysis of Fig. 1. PL peak intensity as a function of precursor ratios (A); Peak position of the relative to the loading wells as a function of precursor ratios (B); Intensity ratios of peaks 1 and 2 as a function of precursor ratios (C); Half width at half maximum (HWHM) of peaks 1 and 2 as a function of precursor ratios (D).


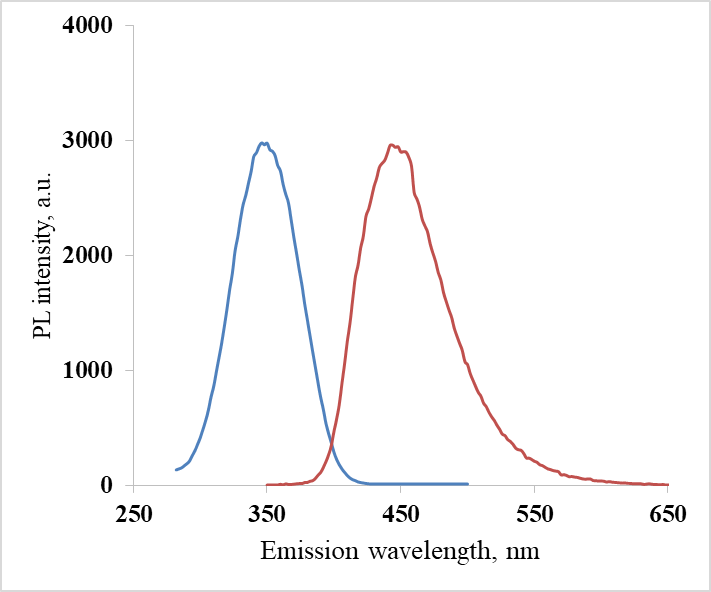

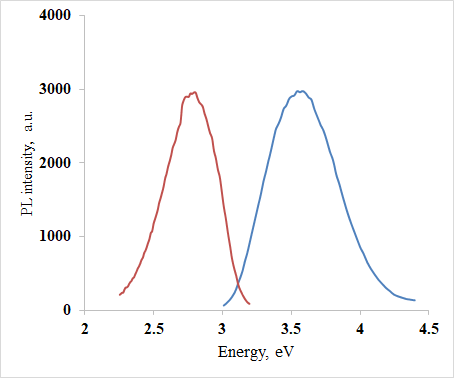


B

A

Fig. S4. Excitation (blue, λ_em_ = 450 nm) and PL spectra (red, λ_ex_  = 350 nm) of HT treated initial solution (CA:EDA = 1:1.5) (A). Symmetry of the excitation and emission peaks is particularly clear on the energy scale (B).


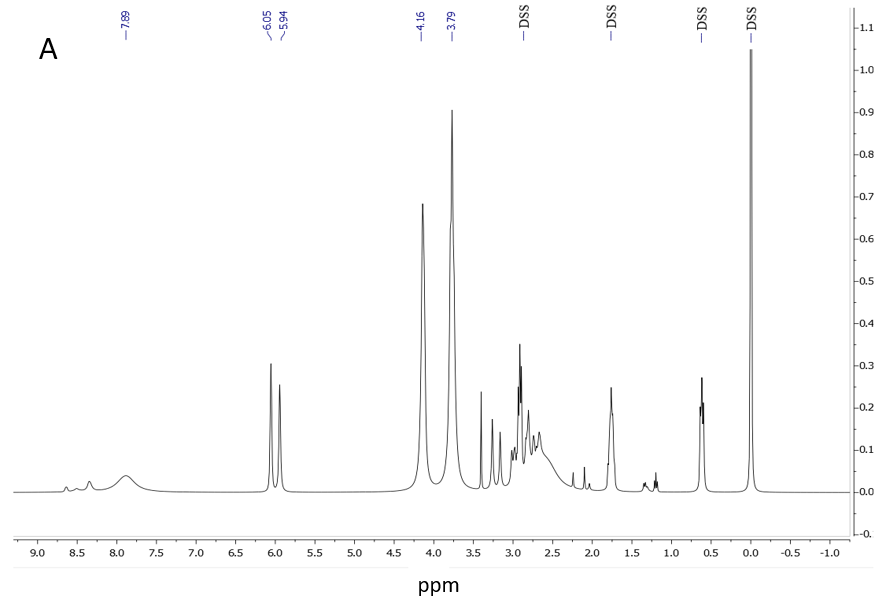

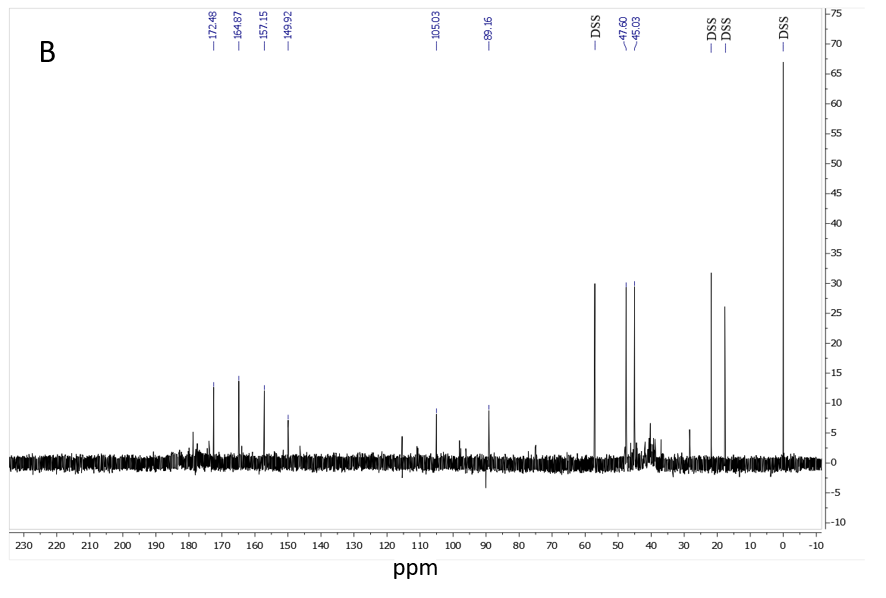

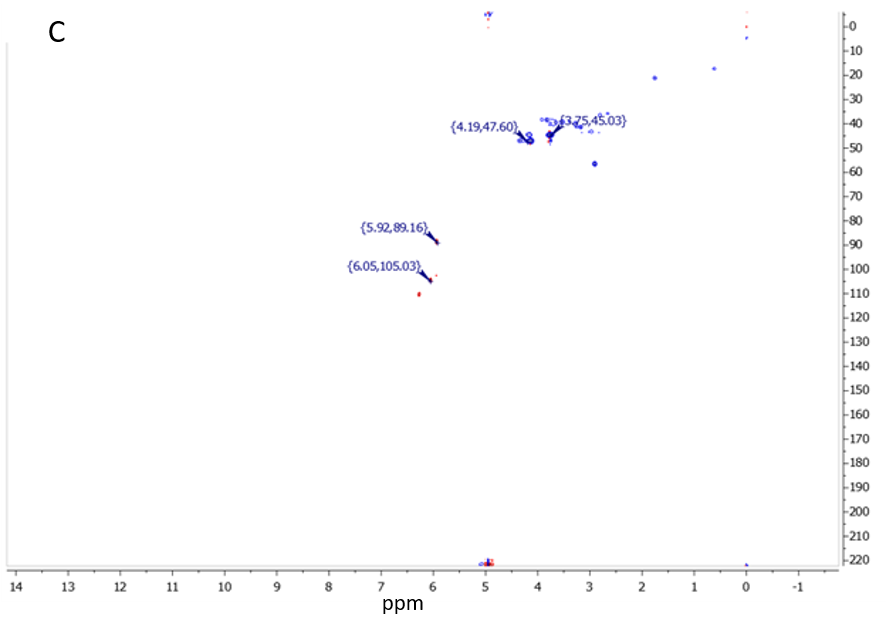


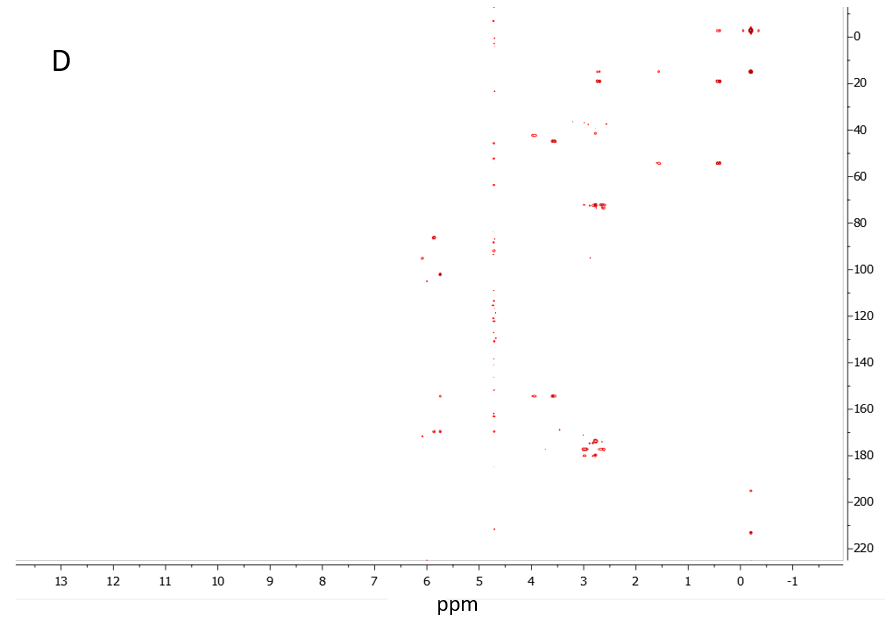


Fig. S5. ^1^H-NMR (A), ^13^С-NMR (B), Heteronuclear single quantum coherence spectroscopy, HSQC (С), Heteronuclear multiple-bond correlation spectroscopy, HMBC (D) spectra of sample СA and EDC with the molar ratio 1:1.5.











D

C

B

A

Fig. S6. Typical TEM images after gel electrophoresis for bands 1 (A), 2(B), 3(C) and 4(D).


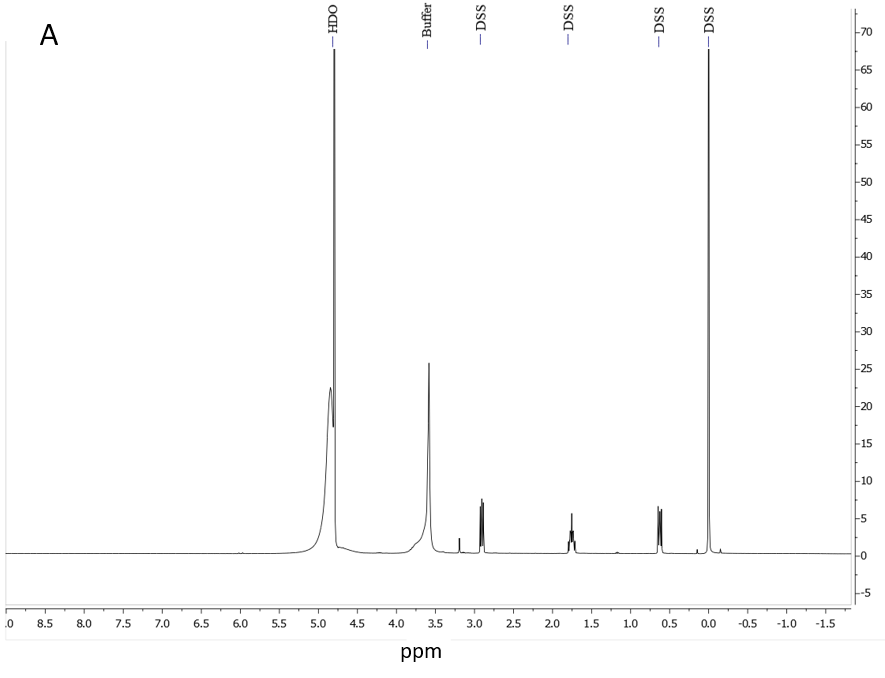

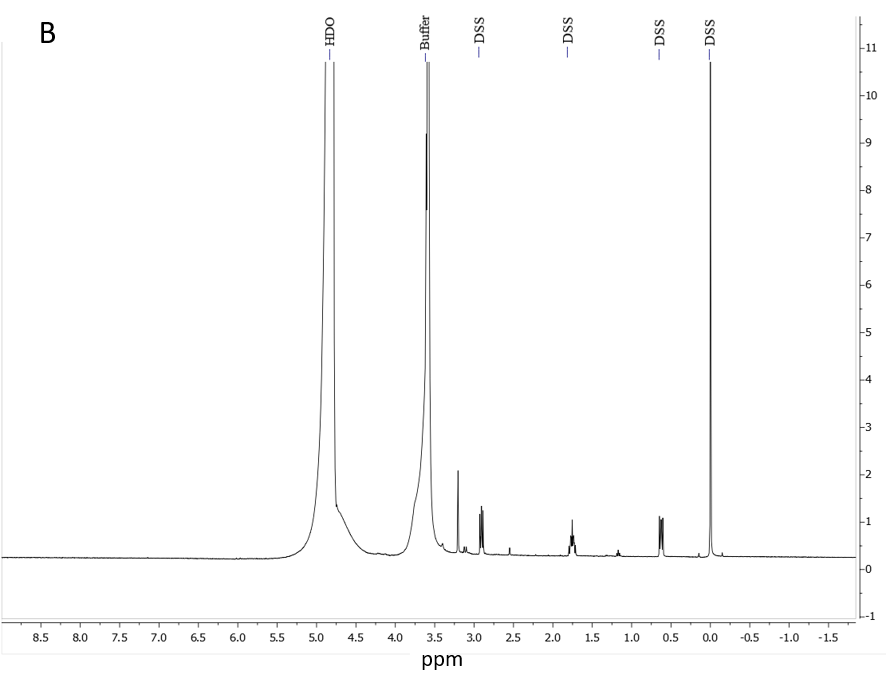

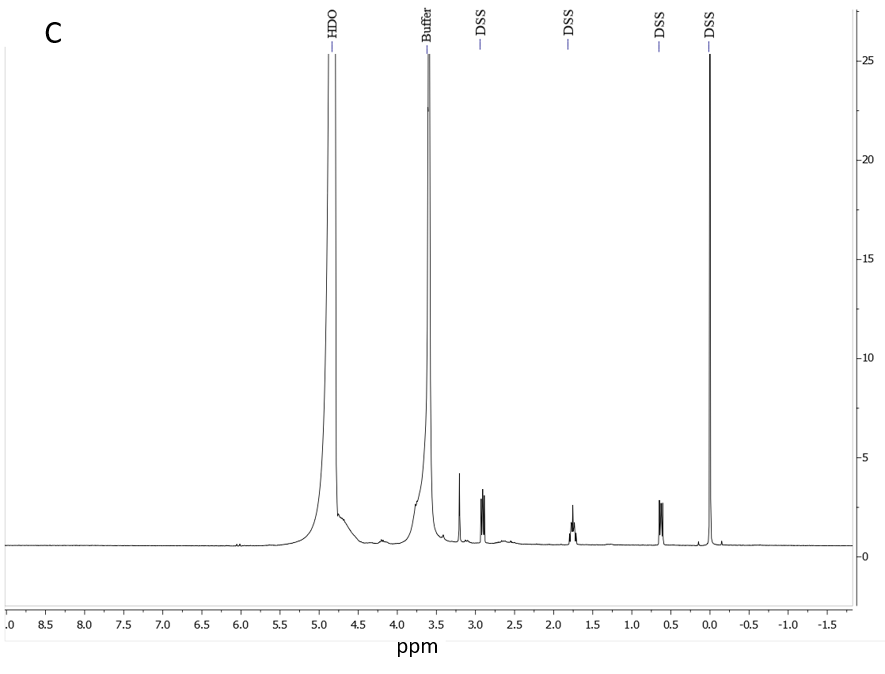

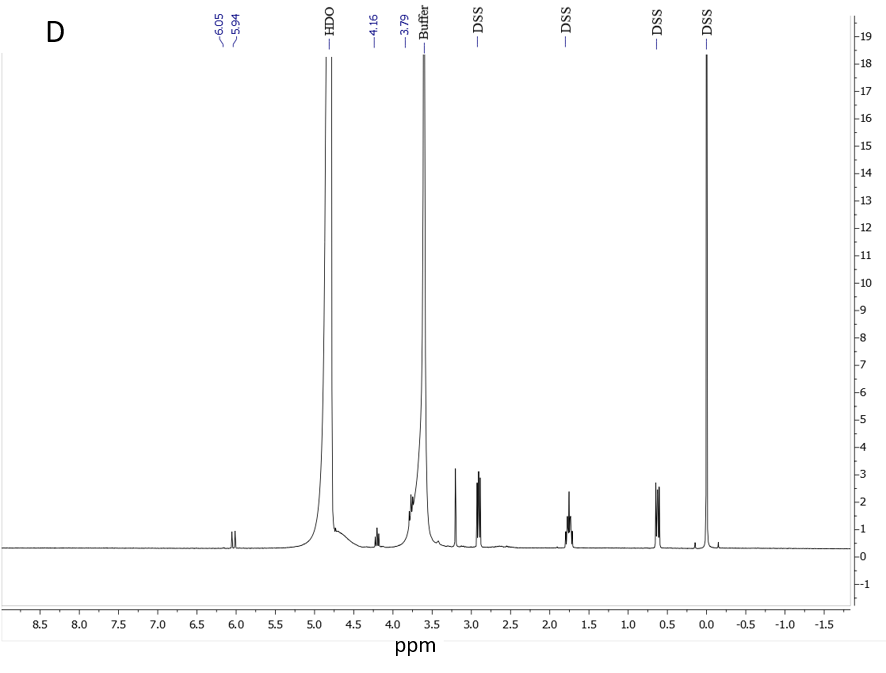


Fig. S7. ^1^H-NMR of gel-separated samples: band 1 (A), band 2 (B), band 3 (C) and band 4 (D). The 1H-NMR spectra of bands 1-3 have a very low intensity of the IPCA signals because of the low concentration of the samples after extraction from the gel. Band 4 contains a sufficient amount of a sample and allow to measure the spectrum that shows IPCA fluorophore signals without polymer-like structure signals.

REFERENCES:

1. Schindelin, J. *et al.* Fiji: An open-source platform for biological-image analysis. *Nat. Methods* **9**, 676–682 (2012).
